# *tert*-Butyl 2-(1*H*-imidazol-1-yl)acetate

**DOI:** 10.1107/S1600536812001067

**Published:** 2012-01-18

**Authors:** Nassir N. Al-Mohammed, Yatimah Alias, Zanariah Abdullah, Hamid Khaledi

**Affiliations:** a Department of Chemistry, University of Malaya, 50603 Kuala Lumpur, Malaysia

## Abstract

In the title compound, C_9_H_14_N_2_O_2_, the imidazole ring and the acetate O—C=O plane make a dihedral angle of 80.54 (12)°. In the crystal, mol­ecules are connected *via* pairs of C—H⋯O hydrogen bonds, forming centrosymmetric dimers.

## Related literature

For related structures, see: Pak *et al.* (2003 ▶); Wang *et al.* (2010 ▶).
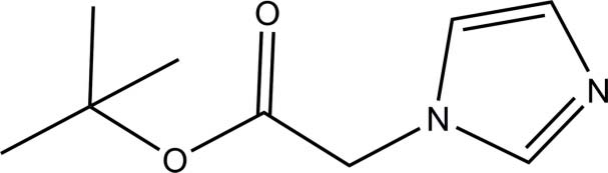


## Experimental

### 

#### Crystal data

C_9_H_14_N_2_O_2_
*M* *_r_* = 182.22
Monoclinic, 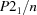

*a* = 10.558 (2) Å
*b* = 9.287 (2) Å
*c* = 11.047 (2) Å
β = 117.157 (4)°
*V* = 963.9 (4) Å^3^
*Z* = 4
Mo *K*α radiation
μ = 0.09 mm^−1^
*T* = 100 K
0.33 × 0.27 × 0.05 mm

#### Data collection

Bruker APEXII CCD diffractometer
Absorption correction: multi-scan (*SADABS*; Sheldrick, 1996 ▶) *T*_min_ = 0.971, *T*_max_ = 0.996
6535 measured reflections
2208 independent reflections
1688 reflections with *I* > 2σ(*I*)
*R*_int_ = 0.035

#### Refinement

*R*[*F* ^2^ > 2σ(*F* ^2^)] = 0.040
*wR*(*F* ^2^) = 0.097
*S* = 1.05
2208 reflections
121 parameters
H-atom parameters constrained
Δρ_max_ = 0.23 e Å^−3^
Δρ_min_ = −0.27 e Å^−3^

### 

Data collection: *APEX2* (Bruker, 2007 ▶); cell refinement: *SAINT* (Bruker, 2007 ▶); data reduction: *SAINT*; program(s) used to solve structure: *SHELXS97* (Sheldrick, 2008 ▶); program(s) used to refine structure: *SHELXL97* (Sheldrick, 2008 ▶); molecular graphics: *X-SEED* (Barbour, 2001 ▶); software used to prepare material for publication: *SHELXL97* and *publCIF* (Westrip, 2010 ▶).

## Supplementary Material

Crystal structure: contains datablock(s) I, global. DOI: 10.1107/S1600536812001067/is5051sup1.cif

Structure factors: contains datablock(s) I. DOI: 10.1107/S1600536812001067/is5051Isup2.hkl

Additional supplementary materials:  crystallographic information; 3D view; checkCIF report

## Figures and Tables

**Table 1:** Hydrogen-bond geometry (Å, °)

*D*—H⋯*A*	*D*—H	H⋯*A*	*D*⋯*A*	*D*—H⋯*A*
C4—H4*B*⋯O2^i^	0.99	2.56	3.3768 (18)	140

Symmetry code: (i) 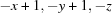.

**Fig. 1.**
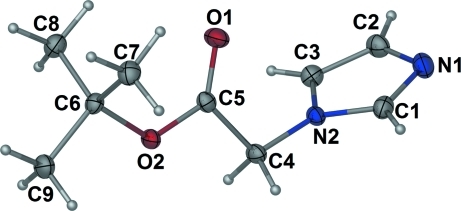
The molecular structure of the title compound showing displacement ellipsoids at the 50% probability level. Hydrogen atoms are drawn as spheres of arbitrary radius.

## supplementary crystallographic information

### Comment

The title compound was obtained through the condensation reaction of imidazole with *tert*-butyl chloroacetate. The imidazole ring and the plane passing through C5/O1/O2, make a dihedral angle of 80.54 (12)°. This value is comparable to those calculated for some similar structures (Pak *et al.*, 2003; Wang *et al.*, 2010). In the crystal, each two molecules are connected *via* a pair of C—H···O bonds around a center of inversion.

### Experimental

Sodium hydroxide (1.32 g, 0.033 mol) was added to a solution of imidazole (1.5 g, 0.022 mol) in DMF (20 ml), followed by addition of *tert*-butyl chloroacetate (3.15 ml, 0.022 mol). The mixture was refluxed for 1 h. The reaction mass was quenched with cold water (50 ml) and extracted by dichloromethane (3 × 25 ml). The combined organic layers was washed with cold water and brine and dried over anhydrous sodium sulfate. The solvent was evaporated under vacuum and the formed amorphous solid was stirred in *n*-hexane (30 ml) at room temperature. The solid was filtered, washed with hexane (2 × 20 ml), and recrystallized from ethyl acetate to afford off-white crystals of the title compound (melting point = 384–386 K).

### Refinement

H atoms were placed at calculated positions and refined in riding mode, with C—H distances of 0.95 (imidazole), 0.98 (methyl) and 0.99 (methylene) Å, and with *U*_iso_(H) set to 1.2 (1.5 for methyl) *U*_eq_(C).

### Crystal data

**Table:**

C_9_H_14_N_2_O_2_	*F*(000) = 392
*M**_r_* = 182.22	*D*_x_ = 1.256 Mg m^−^^3^
Monoclinic, *P*2_1_/*n*	Mo *K*α radiation, λ = 0.71073 Å
Hall symbol: -P 2yn	Cell parameters from 771 reflections
*a* = 10.558 (2) Å	θ = 3.0–29.0°
*b* = 9.287 (2) Å	µ = 0.09 mm^−^^1^
*c* = 11.047 (2) Å	*T* = 100 K
β = 117.157 (4)°	Plate, colorless
*V* = 963.9 (4) Å^3^	0.33 × 0.27 × 0.05 mm
*Z* = 4	

### Data collection

**Table:**

Bruker APEXII CCD diffractometer	2208 independent reflections
Radiation source: fine-focus sealed tube	1688 reflections with *I* > 2σ(*I*)
graphite	*R*_int_ = 0.035
φ and ω scans	θ_max_ = 27.5°, θ_min_ = 2.2°
Absorption correction: multi-scan (*SADABS*; Sheldrick, 1996)	*h* = −10→13
*T*_min_ = 0.971, *T*_max_ = 0.996	*k* = −12→12
6535 measured reflections	*l* = −14→11

### Refinement

**Table:**

Refinement on *F*^2^	Primary atom site location: structure-invariant direct methods
Least-squares matrix: full	Secondary atom site location: difference Fourier map
*R*[*F*^2^ > 2σ(*F*^2^)] = 0.040	Hydrogen site location: inferred from neighbouring sites
*wR*(*F*^2^) = 0.097	H-atom parameters constrained
*S* = 1.05	*w* = 1/[σ^2^(*F*_o_^2^) + (0.0352*P*)^2^ + 0.2973*P*] where *P* = (*F*_o_^2^ + 2*F*_c_^2^)/3
2208 reflections	(Δ/σ)_max_ < 0.001
121 parameters	Δρ_max_ = 0.23 e Å^−^^3^
0 restraints	Δρ_min_ = −0.27 e Å^−^^3^

### Special details

**Table:**

Geometry. All e.s.d.'s (except the e.s.d. in the dihedral angle between two l.s. planes) are estimated using the full covariance matrix. The cell e.s.d.'s are taken into account individually in the estimation of e.s.d.'s in distances, angles and torsion angles; correlations between e.s.d.'s in cell parameters are only used when they are defined by crystal symmetry. An approximate (isotropic) treatment of cell e.s.d.'s is used for estimating e.s.d.'s involving l.s. planes.
Refinement. Refinement of *F*^2^ against ALL reflections. The weighted *R*-factor *wR* and goodness of fit *S* are based on *F*^2^, conventional *R*-factors *R* are based on *F*, with *F* set to zero for negative *F*^2^. The threshold expression of *F*^2^ > σ(*F*^2^) is used only for calculating *R*-factors(gt) *etc*. and is not relevant to the choice of reflections for refinement. *R*-factors based on *F*^2^ are statistically about twice as large as those based on *F*, and *R*- factors based on ALL data will be even larger.

### Fractional atomic coordinates and isotropic or equivalent isotropic displacement parameters (Å^2^)

**Table:**

	*x*	*y*	*z*	*U*_iso_*/*U*_eq_	
O1	0.58413 (11)	0.82030 (11)	0.21337 (11)	0.0244 (3)	
O2	0.42983 (10)	0.63596 (10)	0.11631 (10)	0.0177 (2)	
N1	1.01459 (13)	0.74927 (14)	0.30780 (13)	0.0250 (3)	
N2	0.79333 (12)	0.67798 (12)	0.17766 (12)	0.0164 (3)	
C1	0.91215 (15)	0.66189 (16)	0.29694 (15)	0.0210 (3)	
H1	0.9207	0.5949	0.3653	0.025*	
C2	0.95672 (16)	0.82623 (16)	0.18788 (16)	0.0231 (3)	
H2	1.0055	0.8989	0.1650	0.028*	
C3	0.82115 (15)	0.78441 (15)	0.10713 (15)	0.0197 (3)	
H3	0.7584	0.8211	0.0198	0.024*	
C4	0.65738 (15)	0.60761 (15)	0.13621 (15)	0.0188 (3)	
H4A	0.6720	0.5166	0.1876	0.023*	
H4B	0.6155	0.5835	0.0382	0.023*	
C5	0.55466 (15)	0.70313 (15)	0.16122 (14)	0.0169 (3)	
C6	0.30955 (14)	0.69930 (15)	0.13399 (14)	0.0178 (3)	
C7	0.35240 (16)	0.71586 (17)	0.28396 (15)	0.0228 (3)	
H7A	0.3916	0.6247	0.3308	0.034*	
H7B	0.2688	0.7420	0.2957	0.034*	
H7C	0.4245	0.7916	0.3225	0.034*	
C8	0.26438 (16)	0.84140 (16)	0.05766 (16)	0.0230 (3)	
H8A	0.3393	0.9133	0.1028	0.035*	
H8B	0.1761	0.8747	0.0572	0.035*	
H8C	0.2487	0.8278	−0.0362	0.035*	
C9	0.19439 (15)	0.58562 (16)	0.06881 (15)	0.0230 (3)	
H9A	0.1688	0.5775	−0.0281	0.034*	
H9B	0.1102	0.6134	0.0790	0.034*	
H9C	0.2299	0.4927	0.1135	0.034*	

### Atomic displacement parameters (Å^2^)

**Table:**

	*U*^11^	*U*^22^	*U*^33^	*U*^12^	*U*^13^	*U*^23^
O1	0.0231 (6)	0.0202 (5)	0.0320 (6)	−0.0045 (4)	0.0144 (5)	−0.0086 (4)
O2	0.0141 (5)	0.0182 (5)	0.0222 (5)	−0.0015 (4)	0.0096 (4)	−0.0035 (4)
N1	0.0185 (7)	0.0306 (7)	0.0238 (7)	−0.0012 (5)	0.0077 (6)	−0.0052 (6)
N2	0.0143 (6)	0.0174 (6)	0.0177 (6)	−0.0016 (5)	0.0075 (5)	−0.0027 (5)
C1	0.0195 (7)	0.0230 (7)	0.0192 (7)	0.0036 (6)	0.0076 (6)	0.0006 (6)
C2	0.0199 (7)	0.0233 (7)	0.0290 (8)	−0.0039 (6)	0.0138 (7)	−0.0026 (6)
C3	0.0213 (8)	0.0209 (7)	0.0191 (7)	0.0001 (6)	0.0111 (6)	0.0010 (6)
C4	0.0159 (7)	0.0177 (7)	0.0241 (8)	−0.0026 (5)	0.0102 (6)	−0.0037 (6)
C5	0.0163 (7)	0.0188 (7)	0.0148 (7)	−0.0015 (5)	0.0064 (6)	0.0003 (5)
C6	0.0147 (7)	0.0191 (7)	0.0210 (7)	0.0024 (5)	0.0094 (6)	−0.0002 (6)
C7	0.0218 (8)	0.0268 (8)	0.0216 (8)	0.0020 (6)	0.0116 (6)	−0.0015 (6)
C8	0.0216 (8)	0.0222 (7)	0.0259 (8)	0.0028 (6)	0.0114 (7)	0.0028 (6)
C9	0.0165 (8)	0.0241 (7)	0.0288 (8)	−0.0011 (6)	0.0107 (7)	−0.0011 (6)

### Geometric parameters (Å, °)

**Table:**

O1—C5	1.2039 (17)	C4—H4B	0.9900
O2—C5	1.3325 (16)	C6—C7	1.513 (2)
O2—C6	1.4910 (16)	C6—C9	1.5207 (19)
N1—C1	1.3131 (19)	C6—C8	1.5209 (19)
N1—C2	1.379 (2)	C7—H7A	0.9800
N2—C1	1.3503 (18)	C7—H7B	0.9800
N2—C3	1.3709 (18)	C7—H7C	0.9800
N2—C4	1.4482 (17)	C8—H8A	0.9800
C1—H1	0.9500	C8—H8B	0.9800
C2—C3	1.353 (2)	C8—H8C	0.9800
C2—H2	0.9500	C9—H9A	0.9800
C3—H3	0.9500	C9—H9B	0.9800
C4—C5	1.5208 (19)	C9—H9C	0.9800
C4—H4A	0.9900		
			
C5—O2—C6	121.56 (10)	O2—C6—C9	102.05 (11)
C1—N1—C2	104.32 (12)	C7—C6—C9	111.34 (12)
C1—N2—C3	106.85 (12)	O2—C6—C8	109.88 (11)
C1—N2—C4	127.15 (12)	C7—C6—C8	112.35 (12)
C3—N2—C4	125.75 (12)	C9—C6—C8	111.06 (12)
N1—C1—N2	112.36 (13)	C6—C7—H7A	109.5
N1—C1—H1	123.8	C6—C7—H7B	109.5
N2—C1—H1	123.8	H7A—C7—H7B	109.5
C3—C2—N1	110.88 (13)	C6—C7—H7C	109.5
C3—C2—H2	124.6	H7A—C7—H7C	109.5
N1—C2—H2	124.6	H7B—C7—H7C	109.5
C2—C3—N2	105.60 (13)	C6—C8—H8A	109.5
C2—C3—H3	127.2	C6—C8—H8B	109.5
N2—C3—H3	127.2	H8A—C8—H8B	109.5
N2—C4—C5	111.43 (11)	C6—C8—H8C	109.5
N2—C4—H4A	109.3	H8A—C8—H8C	109.5
C5—C4—H4A	109.3	H8B—C8—H8C	109.5
N2—C4—H4B	109.3	C6—C9—H9A	109.5
C5—C4—H4B	109.3	C6—C9—H9B	109.5
H4A—C4—H4B	108.0	H9A—C9—H9B	109.5
O1—C5—O2	126.65 (13)	C6—C9—H9C	109.5
O1—C5—C4	124.35 (13)	H9A—C9—H9C	109.5
O2—C5—C4	108.99 (11)	H9B—C9—H9C	109.5
O2—C6—C7	109.68 (11)		

### Hydrogen-bond geometry (Å, °)

**Table:**

*D*—H···*A*	*D*—H	H···*A*	*D*···*A*	*D*—H···*A*
C4—H4B···O2^i^	0.99	2.56	3.3768 (18)	140

Symmetry codes: (i) −*x*+1, −*y*+1, −*z*.

## References

[bb1] Barbour, L. J. (2001). *J. Supramol. Chem.* **1**, 189–191.

[bb2] Bruker (2007). *APEX2* and *SAINT* Bruker AXS Inc., Madison, Wisconsin, USA.

[bb3] Pak, J. K., Benny, P., Spingler, B., Ortner, K. & Alberto, R. (2003). *Chem. Eur. J.* **9**, 2053–2061. 10.1002/chem.200204445 12740853

[bb4] Sheldrick, G. M. (1996). *SADABS* University of Göttingen, Germany.

[bb5] Sheldrick, G. M. (2008). *Acta Cryst.* A**64**, 112–122. 10.1107/S0108767307043930 18156677

[bb6] Wang, H.-Y., Zou, P., Xie, M.-H., He, Y.-J. & Wu, J. (2010). *Acta Cryst.* E**66**, o2606. 10.1107/S1600536810037098 PMC2983319 21587583

[bb7] Westrip, S. P. (2010). *J. Appl. Cryst.* **43**, 920–925.

